# Embedding “Smart” Disease Coding Within Routine Electronic Medical Record Workflow: Prospective Single-Arm Trial

**DOI:** 10.2196/16764

**Published:** 2020-07-27

**Authors:** Dee Mangin, Jennifer Lawson, Krzysztof Adamczyk, Dale Guenter

**Affiliations:** 1 Department of Family Medicine McMaster University Hamilton, ON Canada

**Keywords:** chronic disease management, comorbidity, problem list, disease coding, disease registry, data improvement, electronic medical record, electronic health record, practice-based research network, population health, primary care, family medicine

## Abstract

**Background:**

Electronic medical record (EMR) chronic disease measurement can help direct primary care prevention and treatment strategies and plan health services resource management. Incomplete data and poor consistency of coded disease values within EMR problem lists are widespread issues that limit primary and secondary uses of these data. These issues were shared by the McMaster University Sentinel and Information Collaboration (MUSIC), a primary care practice-based research network (PBRN) located in Hamilton, Ontario, Canada.

**Objective:**

We sought to develop and evaluate the effectiveness of new EMR interface tools aimed at improving the quantity and the consistency of disease codes recorded within the disease registry across the MUSIC PBRN.

**Methods:**

We used a single-arm prospective trial design with preintervention and postintervention data analysis to assess the effect of the intervention on disease recording volume and quality. The MUSIC network holds data on over 75,080 patients, 37,212 currently rostered. There were 4 MUSIC network clinician champions involved in gap analysis of the disease coding process and in the iterative design of new interface tools. We leveraged terminology standards and factored EMR workflow and usability into a new interface solution that aimed to optimize code selection volume and quality while minimizing physician time burden. The intervention was integrated as part of usual clinical workflow during routine billing activities.

**Results:**

After implementation of the new interface (June 25, 2017), we assessed the disease registry codes at 3 and 6 months (intervention period) to compare their volume and quality to preintervention levels (baseline period). A total of 17,496 International Classification of Diseases, 9th Revision (ICD9) code values were recorded in the disease registry during the 11.5-year (2006 to mid-2017) baseline period. A large gain in disease recording occurred in the intervention period (8516/17,496, 48.67% over baseline), resulting in a total of 26,774 codes. The coding rate increased by a factor of 11.2, averaging 1419 codes per month over the baseline average rate of 127 codes per month. The proportion of preferred ICD9 codes increased by 17.03% in the intervention period (11,007/17,496, 62.91% vs 7417/9278, 79.94%; *χ*^2^_1_=819.4; *P*<.001). A total of 45.03% (4178/9278) of disease codes were entered by way of the new screen prompt tools, with significant increases between quarters (Jul-Sep: 2507/6140, 40.83% vs Oct-Dec: 1671/3148, 53.08%; *χ*^2^_1_=126.2; *P*<.001).

**Conclusions:**

The introduction of clinician co-designed, workflow-embedded disease coding tools is a very effective solution to the issues of poor disease coding and quality in EMRs. The substantial effectiveness in a routine care environment demonstrates usability, and the intervention detail described here should be generalizable to any setting. Significant improvements in problem list coding within primary care EMRs can be realized with minimal disruption to routine clinical workflow.

## Introduction

Primary care is at the center of health care delivery and coordination and is critically positioned to achieve better population health outcomes and address health inequity within clinical care [1,2]. Chronic disease and multimorbidity are increasingly prevalent in primary care populations [3-6]. Chronic disease identification at the individual level helps to inform better patient care and flags the potential burden of illness and of patients’ care experience. Chronic disease measurement at the practice and population level can help direct prevention strategies and plan health services resource management [3,4,7,8].

The uptake of electronic medical records (EMRs) internationally is high [9]. In Canada, 83% of primary care physicians are using EMRs [10]. Data within primary care EMRs support care for the individual patient. Aggregated, these data may also support practice-based and population health initiatives to understand, target, and deliver care [11], supporting both epidemiological research and quality improvement [11-13]. The Canadian Primary Care Sentinel Surveillance Network (CPCSSN) is one of several national networks that aggregate EMR data to support this work [7,8,14,15]. However, data completeness and consistency of coded values within EMR problem lists or disease registries limit primary and secondary uses of these data [4,16-20].

Primary care clinicians manage, on average, 3 problems per 10- to 15-minute consultation. They have limited time to devote to clinical encounter tasks and even less time for additional data recording and quality tasks that do not relate to individual patient care workflow [21,22]. Primary care physicians spend around half of their clinic time and 1 to 2 hours of after-clinic work devoted to EMR tasks [21,22]. Administrative tasks, including billing, account for around half of the time spent interacting with the EMR.

Primary care practice-based research networks (PBRNs) are clinician collectives focused on asking and answering research questions relevant to their practice context, often using aggregate, routinely collected EMR data. A PBRN offers an ideal setting to imagine and trial interventions that could improve data quality, while not interrupting clinician workflow.

The McMaster University Sentinel and Information Collaboration (MUSIC) PBRN in Hamilton, Ontario, Canada, contributes deidentified EMR data to the CPCSSN national network. Validated algorithms estimate chronic disease prevalence using disease registry codes, billing codes, and medication data [23]. The MUSIC network showed a low prevalence and variability in disease registry codes in relation to the patient population being served.

Our network has been previously successful in implementing an automated, electronic sentinel influenza reporting program integrated into the EMR [24]. We hypothesized that, if co-designed with clinicians, embedding “smart” disease recording within usual EMR clinical workflow could improve disease registry coding volume and quality without any significant burden for clinicians. In this paper, we describe the design, development, and results of a trial of implementation of disease coding tools within the EMR on disease code volume and consistency.

## Methods

We conducted a pragmatic trial of an intervention aimed at improving the quantity and the consistency of coded disease data recorded within the disease registry across the MUSIC PBRN.

### Setting

The study was set within the MUSIC practice-based research network. The MUSIC network holds data on over 75,080 patients, 37,212 currently rostered, from a broad range of neighborhoods within Hamilton, Ontario, Canada, and the surrounding area. All clinicians use the open source EMR, Open Source Clinical Application and Resources (OSCAR).

### Study Design

We used a single-arm prospective trial design with preintervention and postintervention data analysis to assess the effect of the intervention on disease recording volume and quality.

### Intervention Development

We discussed the project rationale with project stakeholders, including clinicians, clinic executives, and MUSIC network staff, to establish project support. There were 5 key aspects to our intervention development: literature review, as-is state investigation of the EMR interface, user engagement in design, standardization of disease codes, and iterative prototype feedback cycles.

#### Literature Review

We first conducted a nonexhaustive literature review to inform the interface design, noting barriers and facilitators for EMR meaningful use [13,25-28]. Prior research demonstrated the concept of leveraging billing workflow for disease-related data improvement [18] and the disease code morbidities most relevant to primary care [27].

#### As-Is State Investigation

The research team investigated the EMR interface for disease data capture within the OSCAR disease registry and within the billing module. Multiple disease registry issues were flagged, including the poor visibility of disease recording tools, which required side-stepped navigation. International Classification of Diseases, 9th Revision (ICD9) code selection was cumbersome due to nonintuitive term names arranged in a large, flat list that lacked organization.

The billing module is an obligatory part of clinical workflow and requires use of provincially issued diagnostic billing codes. The disease coding component of the billing module was explored for its capacity to be leveraged in disease registry code capture, and challenges to this plan were detected. Similar to the ICD9 coding tools, tools for selecting billing codes lacked clinician-friendly naming, quick-pick lists, or an easy method for search and selection of common conditions. Provincial diagnostic billing codes often lacked specificity, bundling several related conditions together, precluding their use in specific disease identification. Of particular note, the last inputted diagnostic code used to bill the previous patient encounter remained populated in the field, satisfying that portion of the data entry criteria for the billing process and providing little incentive for clinicians to choose the diagnostic code best matched to the current patient encounter.

### User Engagement in Design

We engaged 4 clinicians as project advisors and champions. Semistructured interviews with champions identified issues that were possibly contributing to the low volume of disease registry codes and lack of code consistency; these fell into categories of people (physician users), process (workflow and optimized use), and technology (interface).

Stated issues included lack of awareness of how to optimally use disease coding tools, along with time constraints related to clinical workflows and data collection activities. Champions noted a lack of confidence in optimal code selection for both billing codes and disease registry codes, as coding tools were not well supported with search and retrieval tools or quick-pick lists that featured organized and complete sets of preferred terms presented in clinician-friendly formats. Issues of time inefficiency and workflow redundancy related to the need to separately select ICD9 code values for the disease registry when a billing diagnostic code value is already mandated for creating a billing invoice. Champions also reasoned that a firm clinical diagnosis does not always occur at the patient’s first billed encounter for the problem. Disease registry interface issues identified by physicians echoed many of the same constraints and barriers that researchers noted during the as-is state investigation, including low visibility of the disease registry module within the EMR and its lack of integration within clinical documentation workflow.

#### Standardized Disease Codes

We found that the terminology standard, Clinician-Friendly Pick-List Guide for clinical assessment [29], offered for licensed use from the Canadian Institute for Health Information (CIHI), provided a good basis for composing clinician-friendly, chronic disease quick-pick lists for both the billing diagnostic codes and the disease registry codes. We created a reference table composed of 1:1 matches between provincial diagnostic billing codes and the best equivalent ICD9 code to be leveraged for disease registry code capture in the new interface solution (Multimedia Appendix 1).

#### Iterative Design and Feedback Cycles

We developed wire-framed interface prototypes designed to address clinician-noted EMR interface constraints and to increase integration of the disease registry coding into the routine billing process workflow. We sought prototype feedback from clinical champions on (1) the selection of specific codes and their outward-facing names within quick-pick lists, (2) the interface ease of use and its fit into the clinical documentation workflow, and (3) the comprehensibility of data coding interface inputs, screen prompts, and outputs.

The OSCAR EMR service provider contributed substantially to the development of design features that were mindful of the constraints of the EMR platform. A functioning prototype of the interface solution was hosted on a project server and presented to the larger group of clinician end users, with support by clinical champions. This step allowed for consideration of other important design perspectives that were factored into the final interface solution and training of clinician end users.

### Intervention Description

**Figure 1:**
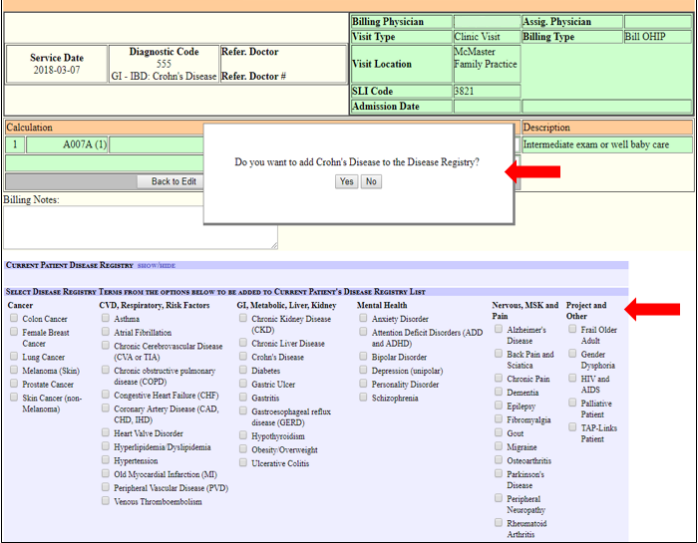
The quick-pick list for disease registry data entry with a pop-up prompt embedded within the billing module.

**Figure 2:**
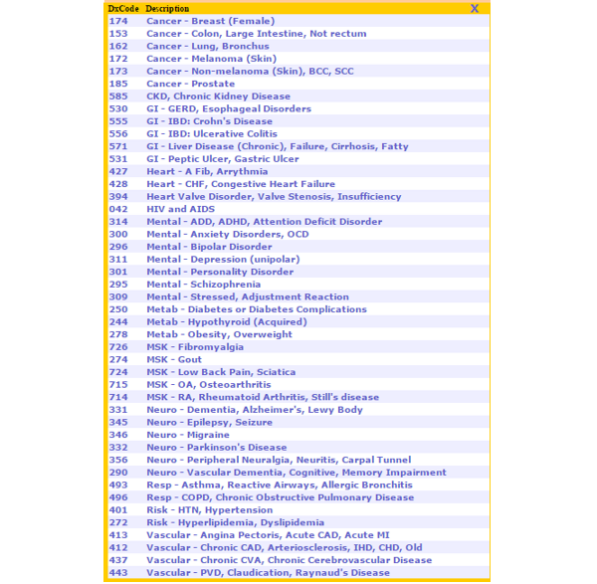
Screenshot of the billing diagnostic quick-pick list.

The final EMR interface solution (Figures 1 and 2) addressed the key issues identified by champions, incorporating disease coding prompts within usual workflow, ease of use, and minimal time burden.

#### Disease Code Quick-Pick Lists

We renamed the ICD disease registry codes with 51 front-facing clinician-friendly terms for common chronic conditions in primary care, guided by the CIHI list and clinical champion feedback. We organized the codes into a quick-pick list with clinically logical groupings and inserted this within the billing module (Figure 1) and the disease registry module. A total of 44 billing diagnostic codes were selected for closest equivalence to the disease registry codes (Multimedia Appendix 1) and fitted with new clinician-friendly term names. Where codes comprised multiple conditions, the one most relevant to the matched ICD9 code formed the leading portion of the term name. These were presented as an easily accessible drop-down list (quick-pick list) within the billing module to be used during obligatory billing activities (Figure 2).

#### Disease Registry Code Prompt Within the Billing Module

The table of billing diagnostic codes matched to ICD9 disease registry codes was posted to the back end of the EMR for automatic nomination of an equivalent disease registry ICD9 code via a pop-up window prompt (Figure 1). The timing of the prompt coincides with clinical cognitive processes around diagnosis and obligatory billing documentation tasks for clinical encounters. When one of the quick-pick billing diagnostic codes is selected, a pop-up screen appears that asks, “Do you want to add [term name] to the disease registry?” with “Yes” and “No” button selections. If the matching ICD9 code value is already in the patient’s disease registry, no prompt is presented. Clicking on “Yes” adds the underlying ICD9 code value to the patient’s disease registry. If “No” is clicked and the same billing code for the same patient is selected at a later consultation, the screen prompt is presented again up to 3 times, after which it is no longer presented. This repeated prompt was suggested by the clinician advisors who gave feedback that diagnosis is not always confirmed at the first presentation for a condition and that 3 times offers a reasonable opportunity to select a disease code without creating undue burden or contributing to alert fatigue.

Once the billing module interface changes were implemented, each clinic site hosted group training sessions for clinician end users that reinforced project rationale and described optimized use of new interface features. Clinician champions at each site encouraged and supported their peers in using the new tools. End users provided interface experience feedback to the project team via clinician champions.

### Outcome Measures

#### Primary Outcome

The primary outcome was the change in total number of disease registry codes in the MUSIC data set compared with the expected number estimated from the preintervention period to assess whether the intervention had been successful.

#### Secondary Outcomes

The secondary outcomes were (1) data consistency, assessed by comparing the proportion of ICD9 codes that matched to the preferred codes at baseline and during the 6-month postintervention phase; (2) usability of the new interface coding tools, assessed by comparing counts of the mode by which the new codes were being added (interface prompts versus other means, eg, direct keying in); and (3) patient characteristics, including the number of patients with disease registry codes identified in their records and whether new codes were added to patients’ partially completed disease registries or de novo, to patients’ disease registries with no previous disease code entries.

### Data Collection Period

We implemented the EMR interface changes on June 25, 2017. The preintervention data set includes all disease registry codes added between January 23, 2006, and June 24, 2017 (baseline period). The intervention data set includes all codes collected on or after the implementation date of June 25, 2017 (intervention period).

We compared the baseline period codes to the intervention period codes at 3 and 6 months after initiation of the intervention to assess their volume and quality.

## Results

### Primary Outcome

**Figure 3:**
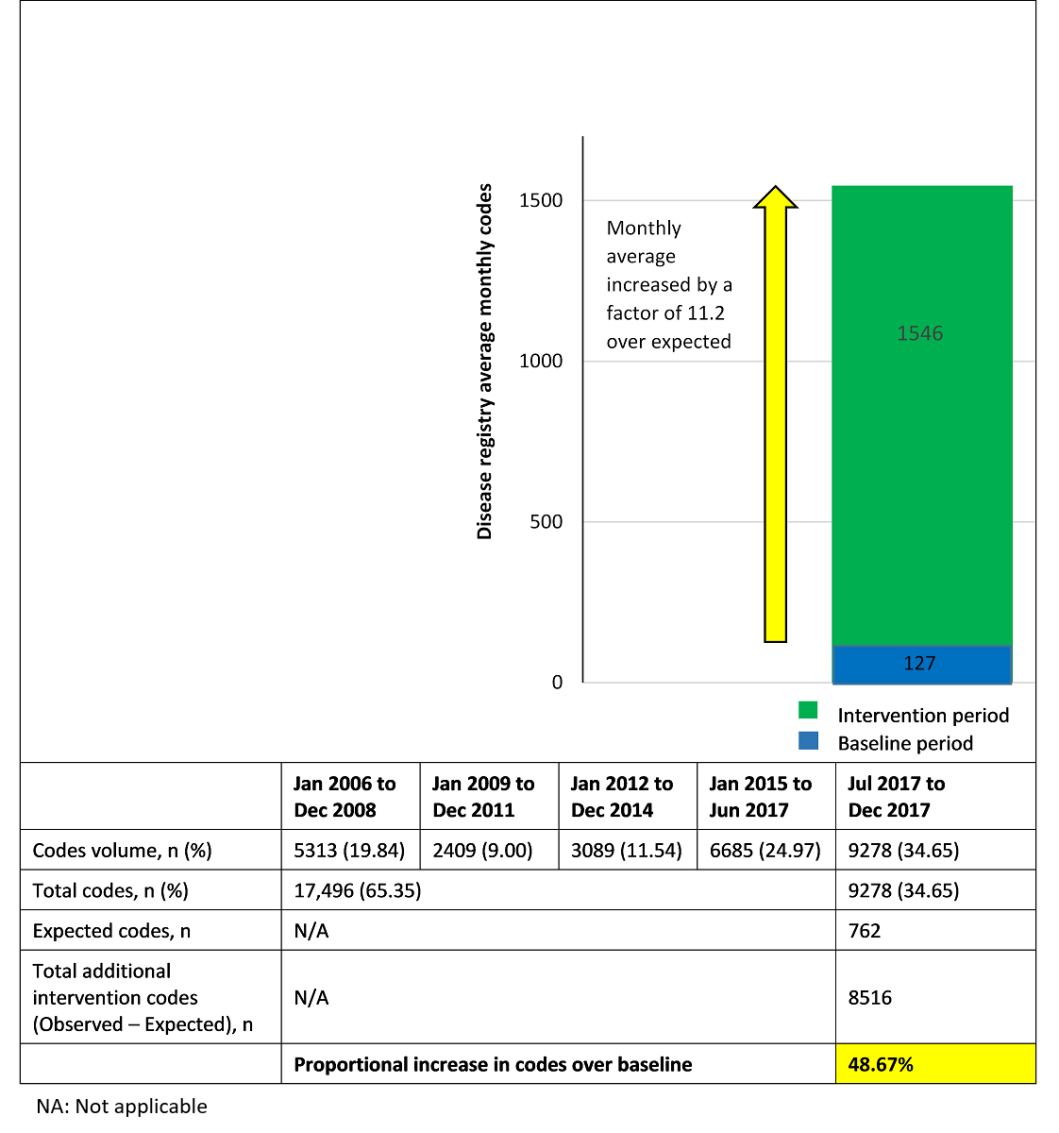
Disease registry monthly code collection rates of baseline and intervention periods.

During the 11-year baseline period (2006 to mid-2017), 17,496 ICD9 code values were recorded in the disease registry. This represents an average code collection rate of 127 codes per month. After implementation of new interface features, 9278 codes were added over 6 months, representing 8516 more codes over the expected volume of 762 codes. Disease registry codes were therefore increased by 48.67% (8516/17,496) by the intervention. The intervention period coding rate averaged 1546 codes per month, which is an increase of 1419 codes per month over the baseline rate (127 codes per month), or a factor of 11.2 (Figure 3). There were more codes added in the first 3 months (6138/9278) of the intervention period compared with the last 3 months (3140/9278).

### Secondary Outcomes

#### Data Consistency

We found a statistically significant percentage point increase of 17.03% (*χ^2^*_1_=819.4; *P*<.001) in the proportion of preferred ICD9 codes selected in the intervention period (7417/9278, 79.94%) compared with the baseline period (11,007/17,496, 62.91%) (Table 1). This shifted the proportion of preferred ICD codes overall from 62.91% (11,007/17,496) to 68.81% (18,424/26,774).

**Table 1:** Proportion of preferred International Classification of Diseases, 9th Revision codes used in the baseline and intervention periods.

Period	Preferred ICD^a^ term codes, n (%) (n=18,424)^b^	Nonpreferred ICD term codes, n (%) (n=8350)^c^	Total codes, n (N=26,774)
Baseline period	11,007 (62.91)	6489 (37.09)	17,496
Postintervention period	7417 (79.94)	1861 (20.06)	9278
Proportional change	3590 (17.03)	4628 (–17.03)	N/A^d^

^a^ICD: International Classification of Diseases.

^b^68.81% of total codes.

^c^31.19% of total codes.

^d^N/A: not applicable.

#### Usability of Coding Tools

Over the 6-month follow-up period, 45.03% (4178/9278) of codes were added via the new screen prompt triggered by the quick-pick list billing codes, with a significant rise in proportion from the first 3 months to the last 3 months (2507/6140, 40.83% vs 1671/3148, 53.08%; *χ^2^*=126.2; *P*<.001). The remaining codes were directly added through (1) the quick-pick list of 51 clinician-friendly disease registry terms positioned within the final screen of the billing module, (2) the quick-pick list in the disease registry module itself, or (3) manually typing the selected codes into the designated field of the disease registry module.

#### Patient Characteristics

A total of 12,459 unique patients had one or more disease registry codes in their record; 28.78% (3486/12,459) had codes recorded during the postintervention period. Among these 3486 patients with postintervention codes, 1527 (43.80%) had no previous disease registry codes in their record, indicating that the new disease coding tools were balanced between extending partially completed disease registries and creating new registries for patients (Multimedia Appendix 1). Demographic characteristics of patients with disease registry coding can be found in Multimedia Appendix 1.

## Discussion

### Principal Results

Our study demonstrates that embedding clinician co-designed EMR disease recording tools into routine workflow, reinforced by training and peer support, results in substantial improvements in the quantity and quality of disease registry coding. In just 6 months, we found an absolute increase of 53.03% (9278/17,496), or a 48.67% (8516/17,496) gain over the number of disease codes expected from the previous 11-year period. There were more codes added in the first 3 months of the intervention period compared with the second 3 months. We saw an increase in the second 3 months in the proportion of codes being added via the new screen prompt triggered by the billing diagnosis code for that encounter. These findings might be expected; the potential gap in disease registry coding narrows as codes are added to a given patient’s problem list for existing but uncoded diseases, so eventually only new disorders identified at subsequent encounters need to be added.

The consistency of codes also increased, with a greater selection of preferred codes added to the disease registry within the intervention period compared with the baseline period. Having a more consistent set of disease codes improves the quality and thereby the value of the data set, supporting both population health research and quality improvement initiatives. The use of the new tools over the older, less systematic ways of entering disease registry codes suggests that this is an acceptable way to substantially increase disease coding and quality.

### Strengths

We used a pragmatic, iterative approach to a primary care EMR enhancement project, with clinician end users involved in design at each step. We applied multiple methods to thoroughly inform the design, including potential solutions from the literature, a national reference standard, and the local EMR service provider. The solution was fitted to routine clinical documentation workflow to limit burden on clinicians. The 6-month follow-up provides a useful and informative assessment of the longitudinal benefit of the intervention. With the pace of change in health informatics, in addition to shifts in definitions for billing codes, gathering follow-up data over this targeted period avoids most potential process and contextual confounders.

### Limitations

While the 6-month evaluation period avoids the confounders highlighted above, it also provides a limited scope with which to measure the long-term success of the interface change. Further longitudinal evaluation will help illuminate any extinction of effect as the coding gap closes and whether the predicted further increase in the overall consistency of codes is supported by the data.

This solution of prompting physicians to add disease registry codes as part of the billing documentation workflow limits coding to patients attending medical appointments. Other solutions for completing the disease registry for patients who attend infrequently will need to be devised to ensure representative problem list data for this group. Disease registry back coding of patients using validated algorithmic case definitions (eg, those offered by CPCSSN [23]) integrated with clinician input may offer a further opportunity to assign missing disease registry codes to inactive patients.

The intervention development and implementation had 5 key aspects of design, as well as training and peer support in implementation. It is not possible to determine the relative contribution of each to the overall effectiveness.

### Comparison With Prior Work

Leading electronic health researchers have identified knowledge and research gaps in primary care EMRs, specifically the need for reliable disease and multimorbidity metrics to inform optimal management of patients’ clinical problems and population-level health strategies [30]. These issues were addressed in this research, first with identification of EMR design constraints affecting disease coding, followed by development, implementation, and evaluation of new data collection tools toward improved data quantity and quality.

Similar to other reported findings [17,19,31,32], we identified data quality issues in the MUSIC EMR data set that limit confidence in the use of chronic disease data for practice-based initiatives and research. Previous research in problem list design identified the benefit of incorporating the problem list into the clinical documentation routine [18,26]; this need was echoed in the feedback from MUSIC clinicians that were consulted in the design of the EMR interface improvement.

EMR usability studies have generated a myriad of clinician observations that identify navigation, safety, and cognitive load issues associated with EMRs [33]. This research underscores the importance of clinician input in EMR design and redesign projects. Continuous engagement of clinician end users in EMR implementation projects [34] or EMR use enhancement projects [35,36] has previously been reported to increase the projects’ likelihood of success [37]. Clinicians in the role of project champions and change management agents have proven essential for the encouragement of advanced EMR feature use [38].

In our study, the application of local physician co-design, which saw key clinician input into solution development, implementation planning, training components, and championing of new coding features, conceivably translated into an interface solution reasonably fitted to clinician workflow, leading to acceptability and uptake. Our study demonstrates that development and delivery of a relevant and usable solution for improving chronic disease recording is attainable.

### Conclusion

Our pragmatic approach to EMR interface redesign resulted in substantial gains in disease code quantity and quality, providing a much-improved data set for asking and answering clinically important research questions. Clinician involvement in the intervention design, training, and peer support resulted in an accepted solution that placed little burden on clinicians. The often used quote, “If we want evidence-based practice, we need practice-based evidence” [39] mandates that PBRN data quality and quantity are adequate for this task. The study demonstrates that achieving significant improvements in problem list coding within primary care EMRs can be realized with minimal disruption to routine clinical workflow.

## Appendix

Multimedia Appendix 1 Supplementary Tables 1-5.

## Abbreviations

CIHI: Canadian Institute for Health Information
CPCSSN: Canadian Primary Care Sentinel Surveillance Network
EMR: electronic medical record
ICD9: International Classification of Diseases, 9th Revision
MUSIC: McMaster University Sentinel and Information Collaboration
OSCAR: Open Source Clinical Application and Resources
PBRN: practice-based research network
