# Supplementary material for: Embedding “Smart” Disease Coding Within Routine Electronic Medical Record Workflow: Prospective Single-Arm Trial
Source: JMIR Med Inform. 2020 Jul 27;8(7):e16764. doi: 10.2196/16764 (PMC7418012; doi:10.2196/16764)
Supplement: Multimedia Appendix 1 [file medinform_v8i7e16764_app1.docx]

## Supplementary Data

### Mapping of Billing Diagnostic Codes to Disease Registry Codes.

Supplementary Table 1. Best matches between billing diagnostic and ICD9 disease registry codes were recorded in a mapped reference table.

| **Billing Diagnostic Code** | **ICD9 disease registry Code** |
| --- | --- |
| 174 | 174 |
| 153 | 153 |
| 162 | 162 |
| 172 | 172 |
| 173 | 173 |
| 185 | 185 |
| 585 | 585 |
| 530 | 530.11 |
| 555 | 555 |
| 556 | 556 |
| 571 | 571 |
| 531 | 531 |
| 427 | 427.3 |
| 428 | 428.0 |
| 394 | 424.9 |
| 042 | 042 |
| 314 | 314.0 |
| 300 | 300.0 |
| 296 | 296.80 |
| 311 | 311 |
| 301 | 301 |
| 295 | 295 |
| 250 | 250 |
| 244 | 244 |
| 278 | 278.0 |
| 726 | 729.1 |
| 274 | 274 |
| 724 | 724 |
| 715 | 715 |
| 714 | 714.0 |
| 331 | 331.0 |
| 345 | 345 |
| 346 | 346 |
| 332 | 332 |
| 356 | 356 |
| 290 | 290 |
| 493 | 493 |
| 496 | 496 |
| 401 | 401 |
| 272 | 272 |
| 413 | 413 |
| 412 | 412 |
| 437 | 437 |
| 443 | 443.9 |
|  |  |

### Analysis of Off-List Codes

We analyzed off-list codes which showed relative high use to inform future extension of the quick-pick-list in a subsequent round of adjustment of disease registry coding tools (Supplementary Table 2).

Supplementary Table 2. Commonly selected ICD9 codes not included in the preferred quick-pick-list.

| **Code** | **OSCAR ICD9 Term Name** |
| --- | --- |
| 733.0 | OSTEOPOROSIS* |
| 790.2 | PREDIABETES |
| 550 | INGUINAL HERNIA* |
| 691 | ATOPIC DERMATITIS* |
| 280 | IRON DEFICIENCY ANEMIAS* |
| 780.51 | INSOMNIA W SLEEP APNEA |
| 366 | CATARACT* |
| 564.1 | IRRITABLE BOWEL SYNDROME |
| 780.57 | OTH UNSPCF SLEEP APNEA |
| 696 | PSORIASIS/LIKE DISORDERS* |
| 354.0 | CARPAL TUNNEL SYNDROME |

Supplementary Table 3. Patients with disease registry codes in baseline and intervention periods.

|  | **Count (%) of patients in disease registry** | **Count (%) of patients with new codes** |
| --- | --- | --- |
| Patients with baseline period codes only | 8,973 (72) |  |
| Patients with baseline and intervention period codes | 1,959 (15.7) | 1,959 (56.2) |
| Patients with intervention period codes only | 1,527 (12.3) | 1,527 (43.8) |
| Total patients with disease registry codes | 12,459 (100) |  |
| Total patients with codes applied during intervention period |  | 3,486 (100) |

### Patient Demographic Characteristics

Supplementary Table 4. Demographic characteristics of patients with disease registry codes in comparison to the entire MUSIC population.

| **Age Bands** | **Patients with disease registry codes (N=12,459), n (%)** | **All MUSIC patients (N=71,707), n (%)** |
| --- | --- | --- |
|  |  |  |
| < 18 |  |  |
|  | 498 (4) | 17,286 (24) |
| 18-25 |  |  |
|  | 642 (5) | 6,335 (9) |
| 26-35 |  |  |
|  | 1,142 (19) | 10,150 (14) |
| 36-45 |  |  |
|  | 1,385 (11) | 9,500 (13) |
| 46-55 |  |  |
|  | 2,001 (16) | 9,086 (13) |
| 56-65 |  |  |
|  | 2,355 (19) | 7,964 (11) |
| 66-75 |  |  |
|  | 2,082 (17) | 4,977 (7) |
| 76-85 |  |  |
|  | 1,270 (10) | 2,706 (4) |
| >85 |  |  |
|  | 1,084 (9) | 3,703 (5) |
| Female |  |  |
|  | 6,992 (56) | 39,344 (55) |
| Male |  |  |
|  | 5,408 (43) | 32,275 (45) |
| Unknown |  |  |
|  | 59 (0) | 88 (0) |
|  |  |  |

The patient population remained relatively stable as measured in the quarter before the intervention (June 2017) and in the intervention period, measured December 2017.

Supplementary Table 5. Stable patient population measured before and after intervention.

|  | June 2017 | Dec 2017 | Delta | Proportional Increase (%) |
| --- | --- | --- | --- | --- |
| MFP Clinic |  |  |  |  |
|  | 34,302 | 34,932 | 630 | 1.8% |
| SFHC Clinic |  |  |  |  |
|  | 36,166 | 37,376 | 1,210 | 3.3% |
| MUSIC Network |  |  |  |  |
|  | 70,468 | 72,308 | 1,840 | 2.6% |
